# A decrease in Fkbp52 alters autophagosome maturation and A152T-tau clearance *in vivo*

**DOI:** 10.3389/fncel.2024.1425222

**Published:** 2024-07-25

**Authors:** Emilie Lesport, Lucie Commeau, Mélanie Genet, Etienne-Emile Baulieu, Marcel Tawk, Julien Giustiniani

**Affiliations:** ^1^Institut Professeur Baulieu, INSERM U1195, Kremlin-Bicêtre, France; ^2^INSERM U1195, Université Paris-Saclay, Kremlin-Bicêtre, France

**Keywords:** tau, tauopathies, lysosomes, autophagy, FKBP52, zebrafish

## Abstract

The failure of the autophagy-lysosomal pathway to clear the pathogenic forms of Tau exacerbates the pathogenesis of tauopathies. We have previously shown that the immunophilin FKBP52 interacts both physically and functionally with Tau, and that a decrease in FKBP52 protein levels is associated with Tau deposition in affected human brains. We have also shown that FKBP52 is physiologically present within the lysosomal system in healthy human neurons and that a decrease in FKBP52 expression alters perinuclear lysosomal positioning and Tau clearance during Tau-induced proteotoxic stress *in vitro*. In this study, we generate a zebrafish *fkbp4* loss of function mutant and show that axonal retrograde trafficking of Lamp1 vesicles is altered in this mutant. Moreover, using our transgenic *HuC::mCherry-EGFP-LC3* line, we demonstrate that the autophagic flux is impaired in *fkbp4* mutant embryos, suggesting a role for Fkbp52 in the maturation of autophagic vesicles. Alterations in both axonal transport and autophagic flux are more evident in heterozygous rather than homozygous *fkbp4* mutants. Finally, taking advantage of the previously described A152T-Tau transgenic fish, we show that the clearance of pathogenic A152T-Tau mutant proteins is slower in *fkbp4*^+/−^ mutants in comparison to *fkbp4*^+/+^ larvae. Altogether, these results indicate that Fkbp52 is required for the normal trafficking and maturation of lysosomes and autophagic vacuoles along axons, and that its decrease is sufficient to hinder the clearance of pathogenic Tau *in vivo*.

## Introduction

Tauopathies are neurodegenerative diseases characterized by abnormal deposition of Tau proteins within the brain of affected patients. In these diseases, including Alzheimer’s disease (AD), Tau becomes hyperphosphorylated and acquires abnormal conformation resulting in the formation of toxic oligomers and intracellular aggregates (Gotz et al., 2019).

The failure of degradative pathways such as the ubiquitin proteasome system or the autophagy-lysosomal pathway (ALP) to clear the pathogenic forms of Tau participates in the pathogenesis of neurodegenerative diseases (Chesser et al., 2013; Fleming et al., 2022). Macro-autophagy (here after named autophagy) is a mechanism by which cytoplasmic compounds become engulfed into double-membraned vesicles called autophagosomes which ultimately fuse with lysosomes to allow cargo degradation (Stavoe and Holzbaur, 2019). In the case of the highly polarized neurons, the maturation of autophagic vacuoles (AVs) is tightly linked with their transport along the axon. Autophagosomes are predominantly formed at the axon tip before being retrogradely transported toward the cell body by dynein motor complex (Maday et al., 2012). During this transport, successive encounters with late endosomes and lysosomes allow the progressive acidification of the autophagosomal lumen and maturation towards degradative autolysosomes (Mathew and Koushika, 2022; Roney et al., 2022).

FKBP52 (FK506-binding protein of 52 kDa) is a member of the immunophilin family coded by the *FKBP4* gene (Lebeau et al., 1992). It presents peptidyl-prolyl *cis-trans* isomerase activity and is implicated in protein folding, stability, and trafficking (Chambraud et al., 2022). FKBP52 is highly abundant within the central nervous system, and interacts both physically and functionally with Tau (Chambraud et al., 2010; Giustiniani et al., 2014). We have previously shown that the level of FKBP52 proteins is strongly reduced in the frontal cortex of patients affected with AD and FTLD-Tau (frontotemporal lobar degeneration with Tau pathology) (Giustiniani et al., 2012). While FKBP52 is detected in the lysosomal system in healthy human brain neurons, residual FKBP52 from diseased patients is often colocalized with AVs (Meduri et al., 2016, 2023). More recently, we have shown that FKBP52 deficiency hinders the correct lysosomal positioning during Tau-induced proteotoxic stress in different neuronal cells. This leads to either Tau secretion or insoluble Tau accumulation depending on the cell phenotype and time-scale of Tau induction (Chambraud et al., 2021). All these data raise the possibility that FKBP52 promotes the autophagic clearance of Tau.

To test whether Fkbp52 activity is required in the ALP *in vivo*, we here use the zebrafish as model system. We generate a *fkbp4* loss of function zebrafish mutant and show an increase in the mean velocity of retrogradely transported Lamp1 vesicles in these mutants. We also find a significant decrease in the percentage of autolysosomes in mutant embryos, suggesting a defective maturation of autophagosomes. Finally, using the previously described *Tg(UAS::Dendra-Tau A152T)* line (Lopez et al., 2017), we observe that the clearance of A152T-Tau is slower in *fkbp4*^+/−^ zebrafish compared to *fkbp4*^+/+^ controls. Altogether, we provide evidence of a role of Fkbp52 in the transport and maturation of vesicles of the autophagic pathway and in the clearance of pathogenic Tau *in vivo*.

## Materials and methods

### Embryo care

Zebrafish were raised and maintained under standard conditions^1^. Embryos were staged according to established criteria (Kimmel et al., 1995) and reared in embryo medium (5 mM NaCl, 0.17 mM KCl, 0.33 mM CaCl_2_, 0.33 mM Mg_2_SO_4_, 5 mM HEPES). All animal experiments were conducted with approved protocols at Inserm by DDPP Val de Marne, France, under license number F 94-043-013.

Wild-type (WT) zebrafish were of AB strain. The stable transgenic lines *Tg(HuC::Gal4)* and *Tg(UAS::Dendra-Tau A152T)* were kind gifts from Herwig Baier, Max Planck Institute for biological intelligence, Martinsried, Germany (Scott et al., 2007) and David C. Rubinsztein, University of Cambridge, Cambridge Institute for medical research, Cambridge, United Kingdom (Lopez et al., 2017), respectively.

### CRISPR-Cas9 mutagenesis

The *fkbp4* mutant lines were obtained using CRISPR-Cas9 mutagenesis as previously described (Mikdache et al., 2021). Briefly, gRNA ‘GGCACCGAGTTGCCTATGAT’ targeting exon 2 of the *Danio rerio fkbp4* gene was designed using CRISPOR website^2^ and generated by cloning free method (Varshney et al., 2015). One-cell stage WT zebrafish zygotes were injected with 300 ng/μL of gRNA and 20 μM Cas9 nuclease (NEB). Injected embryos were raised to adulthood and outcrossed to WT fish. The F1 offspring was screened for the presence of frameshift mutation by sequencing. F0 founders transmitting an allele with a 1 base pair (bp) insertion “+1” or a 11 bp deletion “Δ11” were chosen to establish 2 mutant lines. Heterozygous fish from both lines were outcrossed for at least 3 generations before being incrossed to obtain homozygous fish used in experiments.

To discriminate mutants from WT fish, a locus specific PCR was performed using forward 5′-GCGTGAACATCTTTCTGCCC-3′ and reverse 5’-TTTAAGAGTCAGCGAGGGCG-3′ primers. The PCR product was then digested using restriction enzyme BslI, whose recognition site is disrupted following Cas9-induced mutagenesis.

### Western blot

Fish from mutant clutches were frozen individually at −80°C after cutting their tail for genomic DNA extraction. The tail of WT embryos was also cut to ensure equal treatment with mutant fish. Following genotyping, 15–20 fish of same genotype were pooled and lysed on ice with lysis buffer containing 63 mM Tris HCl pH 6.8, 10% glycerol, 3.5% SDS and protease inhibitor cocktail (Merck). Extraction was performed in glass pestles using Ultra-Turrax homogenizer and protein contents were determined using Pierce BCA protein assay (ThermoFisher). Samples containing 20 μg of proteins were loaded on 10% polyacrylamide gels, then resolved by electrophoresis and further transferred to nitrocellulose membranes. The following antibodies were used: rabbit anti-FKBP4 (Invitrogen #PA5-22277, 1/500 dilution), mouse anti-β-actine (Sigma #1978, 1/2000 dilution), rabbit anti-α-tubuline (Abcam #ab18251, 1/5000 dilution) and HRP-conjugated secondary antibodies (Interchim, 1/10000 dilution). Chemiluminescent signals were obtained using Pierce ELC plus or West Femto substrates (ThermoFisher) and captured with GeneGnome5. Quantifications were performed using ImageJ.

### DNA constructs

To obtain the *pT2-HuC:Lamp1-mEGFP* plasmid, a *lamp1-mEGFP* cassette flanked by SpeI and MluI sites was PCR-amplified with 5′-GAATTCACTAGTATGGCGGCCCCCGGCAG-3′ forward and 5′-TGTATCACGCGTTTACTTGTACAGCTCGTCC-3′ reverse primers using Addgene plasmid #34831 (gift from Esteban Dell’Angelica) (Falcon-Perez et al., 2005) as template. The resulting DNA fragment was subcloned into the *pT2-HuC:Dendra2* plasmid (gift from Periklis Pantazis, Addgene #80904) (Mohr et al., 2016) previously modified to introduce multiple cloning sites 3′ to the Dendra2 sequence, replacing Dendra2. One-cell stage embryos were injected with 10 pg of the *pT2-HuC:Lamp1-mEGFP* plasmid.

To generate the *pT2-HuC:mCherry-EGFP-Lc3* plasmid, the *mCherry-EGFP-map1lc3b* DNA sequence was amplified by PCR assembly using 5’-GATTTAGGTGACACTATAG-3′ forward and 5′-TTTTGTACAAACTTGTTCCTGGGCCAGCGGATCTGAGTCCGGACTTGTACAGCTCGTCCATGC-3′ reverse primers on *pCS2-mCherry* plasmid and 5’-AGGAACAAGTTTGTACAAAAAAGCAGGCTTTCCGGTCGCCACCATGGTGAGCAAGGGCGAG-3′ forward and 5′-TGTATCATGCATTTACTGAAATCCAAATGTCT-3′ reverse primers on genomic DNA extracted from the *Tg(CMV::EGFP-map1lc3b)* fish (He et al., 2009), introducing a 66 bp linker between the *EGFP* and *mCherry* sequences and a NsiI restriction site. The obtained DNA fragment was subcloned into the *pT2-HuC:Dendra2* plasmid using BamHI and PstI to replace *Dendra2*.

### Generation of Tg(HuC::mCherry-EGFP-map1lc3b) line

To generate the *HuC::mCherry-EGFP-map1lc3b* transgenic line, WT zygotes were injected with 20 pg of the *pT2-HuC:mCherry-EGFP-Lc3* plasmid mixed with 100 pg of *in-vitro* transcribed (SP6 mMESSAGE mMACHINE kit, ThermoFisher) Tol2 transposase mRNA. Injected embryos were raised to adulthood and outcrossed to WT fish. The F1 offspring was screened for presence of the transgene by PCR and then for the presence of mCherry-EGFP-Lc3 signal within neurons using Leica SP8 confocal microscope. Out of 48 F0 tested, 5 transmitted the transgene to their germline, but expression of the transgene was only observed in one out of 5 founders. The expression was mosaic with slightly variable numbers of labelled neurons from one embryo to another, and remained stable for at least 5 generations.

F1 *Tg(HuC::mCherry-EGFP-map1lc3b)* fish was crossed with *fkbp4* “+1” mutant fish to observe mCherry-EGFP-Lc3 vesicles in a *fkbp4* mutant background.

### Live imaging

Embryos were first anesthetized using tricaine diluted in embryo medium and then embedded in 1.4% low melting point agarose. Acquisitions were performed at 27°C on Leica SP8 confocal microscope.

To study the movement of lysosomes along axons, one-cell stage embryos were injected with *pT2-HuC:Lamp1-mEGFP* construct and imaged at 48 hpf. Embryos showing isolated positive axons were selected for time-lapse imaging. For each positive embryo, videos were acquired along 2 or 3 segments of the spinal cord using 40x objective and 3x magnification, with a time interval of 650 milliseconds. Individual vesicles were then tracked for at least 10 images using ImageJ ‘Manual tracking’ plugin.

For experiments using *Tg(HuC::mCherry-EGFP-map1lc3b)* fish, embryos were imaged at 48 hpf. A *z*-stack (1 μm interval) was acquired within a specific region of the hindbrain showing axonal projections with a high density of positive vesicles using 40× objective and 3× magnification. For bafilomycin treatment, embryos were incubated overnight with 20 nM of bafilomycin or with the equivalent volume (0.04%) of DMSO as control, before acquisition.

All images and videos shown are from lateral views, anterior to the left, dorsal to the top.

Image analyses were performed using ImageJ. To determine the autophagosomes/autolysosomes ratio, for each optical slice of the acquired stack, mCherry-positive vesicles were spotted manually along axons, and the presence of a EGFP signal was then checked for each vesicle. Red and green vesicles were counted as autophagosomes whereas red-only vesicles were counted as autolysosomes.

When using *fkbp4* mutant embryos, DNA was extracted at the end of each set of acquisitions and genotyping was only performed after completion of the analyses.

### Dendra-tau clearance assays

*Tg(HuC::Gal4)* driver fish and *Tg(UAS::Dendra-Tau A152T)* responder fish were crossed to obtain *fkbp4*^+/+^ offspring with a pan-neuronal expression of A152T-Tau. The *fkbp4* “+1” mutant line was crossed with *Tg(HuC::Gal4) and Tg(UAS::Dendra-Tau A152T)* fish. Resulting *fkbp4*^−/−^ driver or responder females were crossed with *fkbp4*^+/−^ responder or driver males, respectively, to obtain *fkbp4*^+/−^ and *fkbp4*^−/−^ embryos with pan-neuronal expression of A152T-Tau.

At 48 hpf, a segment of 73 μm of the spinal cord was irradiated for 15 s with the UV laser (405 nm) using the 40× objective of the Leica SP8 confocal microscope at 4× magnification to allow photoconversion from Green to Red-Dendra. A *z*-stack of the photoconverted region was acquired just after photoconversion and then 24 h and 48 h later using the same settings. DNA was extracted at the end of each set of acquisitions and genotyping was only performed after completion of the analyses.

The intensity of the red signal of the photoconverted region at each time point was quantified using ImageJ ‘Sum Slices’ *z*-projection of the whole stack and expressed as the percentage of the initial Red-Dendra intensity.

To allow comparison of *fkbp4* WT and mutant embryos with similar levels of A152T-Tau expression, a *z*-stack of the spinal cord was systematically taken at 48 hpf for each embryo under 20× objective, making sure that the laser and detection settings remained always identical. The intensity of the expression was quantified using ImageJ ‘Sum Slices’ *z*-projection of the whole stack. Only embryos with an intensity between 100 and 200 (arbitrary units) were included for analysis.

### RT-qPCR

Fish from mutant clutches were frozen individually at −80°C after cutting their tail for genomic DNA extraction. The tail of WT embryos was also cut to ensure equal treatment with mutant fish. Following genotyping, 15–20 fish of same genotype were pooled in Trizol. RNA extraction and reverse transcription were performed as previously described (Mikdache et al., 2021). Primers are listed in Supplementary Table S1 (Li et al., 2023).

### Statistical analyses

Statistical analyses were performed using GraphPad Prism 7.0. Unpaired Student’s *t*-tests were used when only 2 groups of values with gaussian distribution were compared. For comparison of more than two groups, Anova or Kruskal–Wallis test followed by multiple comparisons test were used. *p*-values lower than 0.05 were considered significant. *p < 0.05, **p < 0.01, ***p < 0.001, and ****p < 0.001.

## Results

### Fkbp52 mutant larvae are viable and have no external phenotype

**Figure 1:**
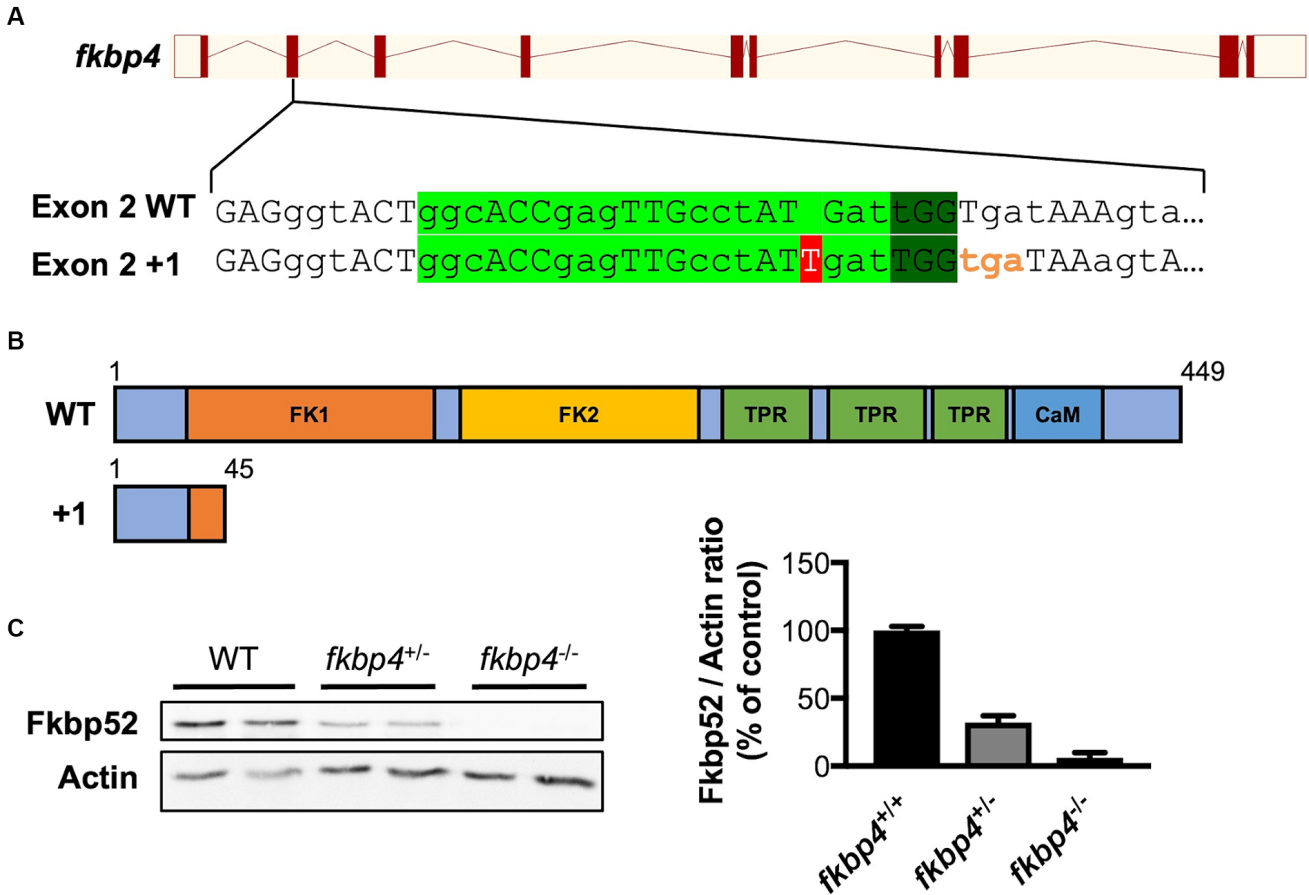
Generation of *fkbp4* mutant zebrafish. **(A)** Position of the CRISPR/Cas9 target site (light green) and PAM sequence (dark green) within exon 2 of the *Danio rerio fkbp4* gene. The 1 bp insertion (red) on the mutant allele induces a frameshift and a premature stop codon (orange). **(B)** Structural domains of the WT Fkbp52 protein and of the “+1” mutant Fkbp52 protein. FK1: peptidyl-prolyl isomerase (PPIase) domain. FK2: PPIase-like domain. TPR: tetratricopeptide repeat domain. CaM: putative calmodulin binding site. **(C)** Representative immunoblot and resulting quantification showing Fkbp52 levels in WT, *fkbp4*^+/−^ and *fkbp4*^−/−^ embryos. Actin was used as loading control.

We have previously shown that FKBP52 is present in the lysosomal system of neurons from human and murine samples and that the activity of the ALP was affected by FKBP52 knock-down *in vitro* (Meduri et al., 2016; Chambraud et al., 2021). To investigate the function of Fkbp52 in autophagy *in vivo*, we chose to use the transparent zebrafish larvae that offer a unique opportunity to monitor the temporal and functional activity of proteins (Boueid et al., 2020). We generated a loss of function mutant of the *Danio rerio fkbp4* gene encoding Fkbp52, using CRISPR/Cas9 technology. The introduced mutation created a frameshift leading to a premature stop codon and the loss of Fkbp52 protein in homozygous embryos as revealed by Western blot (Figure 1).

Homozygous mutant embryos developed normally and were undistinguishable from WT fish. However, as they reached adulthood, *fkbp4*^−/−^ males were mostly sterile while homozygous females were all fertile. As a result, maternal-zygotic *fkbp4*^−/−^ embryos could only be obtained as part of the progeny of homozygous females crossed with heterozygous males, that we hereafter refer to as ‘mutant’ clutches.

### Retrograde transport of Lamp1 vesicles is accelerated in *fkbp4* mutant zebrafish

**Figure 2:**
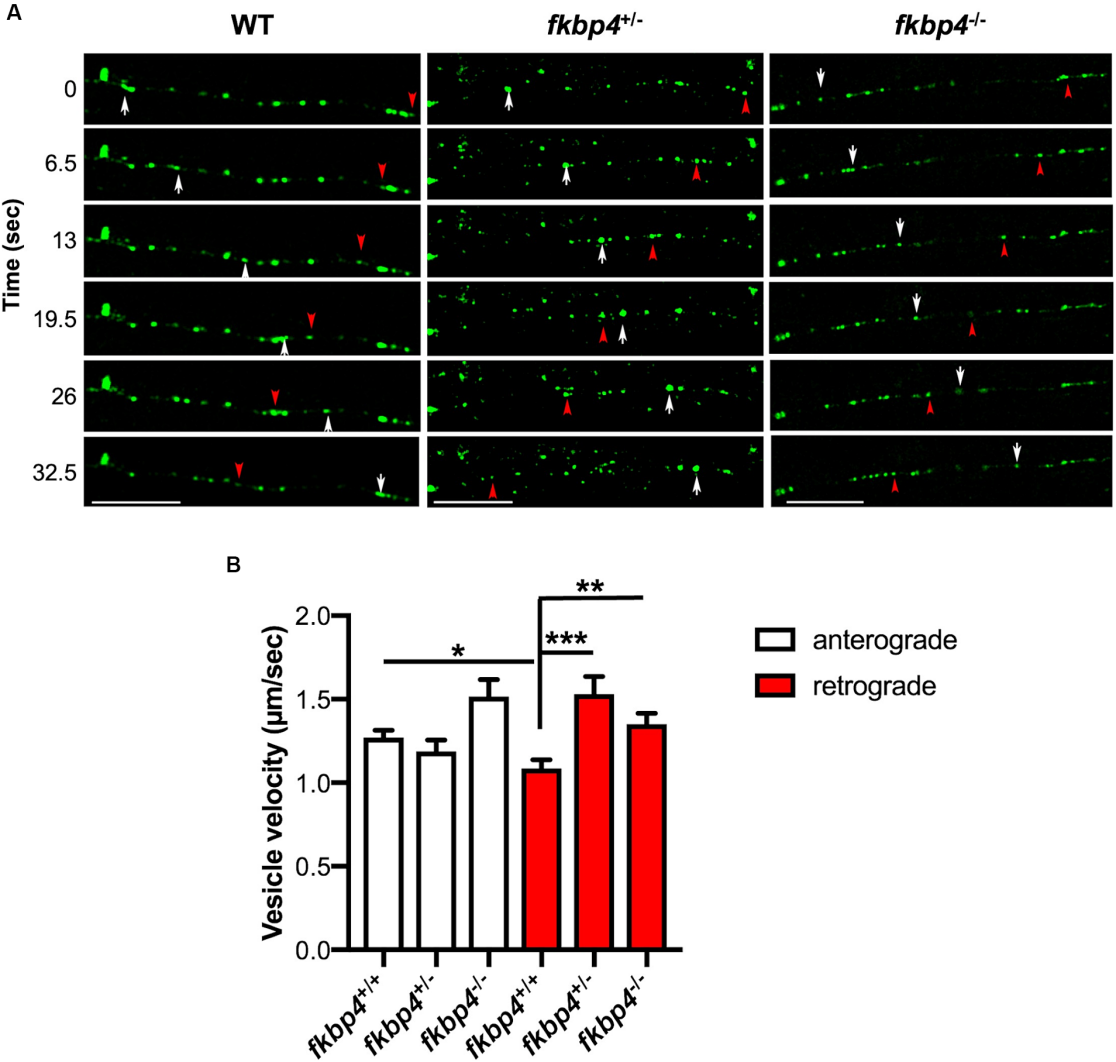
A decrease in Fkbp52 impacts the retrograde velocity of Lamp1-positive vesicles in spinal cord axons. **(A)** Representative still images of time-lapse imaging at 48 hpf in control, *fkbp4*^+/−^ and *fkbp4*^−/−^ embryos injected with *pT2-HuC:Lamp1-mEGFP*; white arrows and red arrowheads delineate the anterograde and retrograde transport of Lamp1-positive vesicles, respectively. Scale bar, 20 μm. **(B)** Quantification of anterograde and retrograde velocity of Lamp1-positive vesicles along axons of spinal cord in WT (*n* = 11), *fkbp4*^+/−^ (*n* = 5) and *fkbp4*^−/−^ (*n* = 6) embryos from 3 independent experiments. Analysis includes ~12 tracked Lamp1 vesicles per embryo (Kruskal–Wallis, Dunn’s multiple comparisons test). Results are shown as mean ± SEM.

It has been shown that FKBP52, in association with the protein chaperone HSP90 and dynein motor, is implicated in the retrograde transport and nuclear translocation of different proteins. This includes the glucocorticoid receptor, the transcription factor p53 and the human telomerase reverse transcriptase hTERT (Galigniana et al., 2004; Wochnik et al., 2005; Jeong et al., 2016). Knowing that the axonal trafficking of autophagic vesicles also relies on dynein motor complex (Maday et al., 2014), we wondered whether Fkbp52 could play a role in their transport along microtubules. We chose to monitor endolysosomal vesicles using Lamp1 as a marker. Zygotes were injected with the *pT2-HuC:Lamp1-mGFP* construct and isolated Lamp1-positive vesicles were tracked along individual axons in the spinal cord at 48 hpf (Figure 2). In WT embryos, as expected, the anterograde transport was significantly faster than the retrograde transport (1.27 μm/s ± 0.044 and 1.0865 μm/s ± 0.051 respectively) (Drerup and Nechiporuk, 2013; Boecker et al., 2020). No significant difference was observed between WT and *fkbp4* mutant embryos regarding the anterograde transport of Lamp1-positive vesicles (1.187 μm/s ± 0.069 and 1.516 μm/s ± 0.101 for *fkbp4*^+/−^ and *fkbp4*^−/−^ respectively). By contrast, we observed a significant increase in the mean velocity of Lamp1-positive vesicles moving retrogradely in *fkbp4*^−/−^ embryos (1.35 μm/s ± 0.066) compared to WT. Unexpectedly, the same alteration of the retrograde transport of Lamp1-positive vesicles was also observed in *fkbp4*^+/−^ embryos (1.531 μm/s ± 0.105). Using a second *fkbp4* mutant fish line that we similarly generated using Cas9-mutagenesis, we also found that the mean retrograde velocity of Lamp1-positive vesicles was significantly increased in heterozygous mutants compared to WT (Supplementary Figure S1).

These results indicate that the pool of Lamp1-positive vesicles moving toward the cell body is affected by a decrease in Fkbp52.

### Autolysosomes are less abundant in neurons of *fkbp4* mutant zebrafish

The regulated transport of lysosomes and AVs along axons is tightly linked to their maturation and any alteration in this process could hinder autophagic flux (Tammineni and Cai, 2017; Mathew and Koushika, 2022).

To monitor the autophagic flux within neurons, we generated a new zebrafish transgenic line expressing the mCherry-EGFP fluorescent tandem fused to Lc3, a marker of AVs, under control of the pan-neuronal promoter HuC. The mCherry-EGFP-Lc3 reporter allowed us to discriminate between autophagosomes which appear as yellow vesicles (mCherry-positive and EGFP-positive) and autolysosomes where only the mCherry fluorescence remains after quenching of the EGFP signal due to the acidification of the vesicular lumen (Kimura et al., 2007).

*Tg(HuC::mCherry-EGFP-Lc3)* embryos, hereafter named *HCGL* embryos, showed a mosaic expression of the transgene, but yellow and red AVs were easily visible and distinguishable in labelled neurons at 48 hpf (Supplementary Figure S2). As expected, cell soma contained virtually only red autolysosomes, whereas both red and yellow AVs were observed along axonal projections.

**Figure 3:**
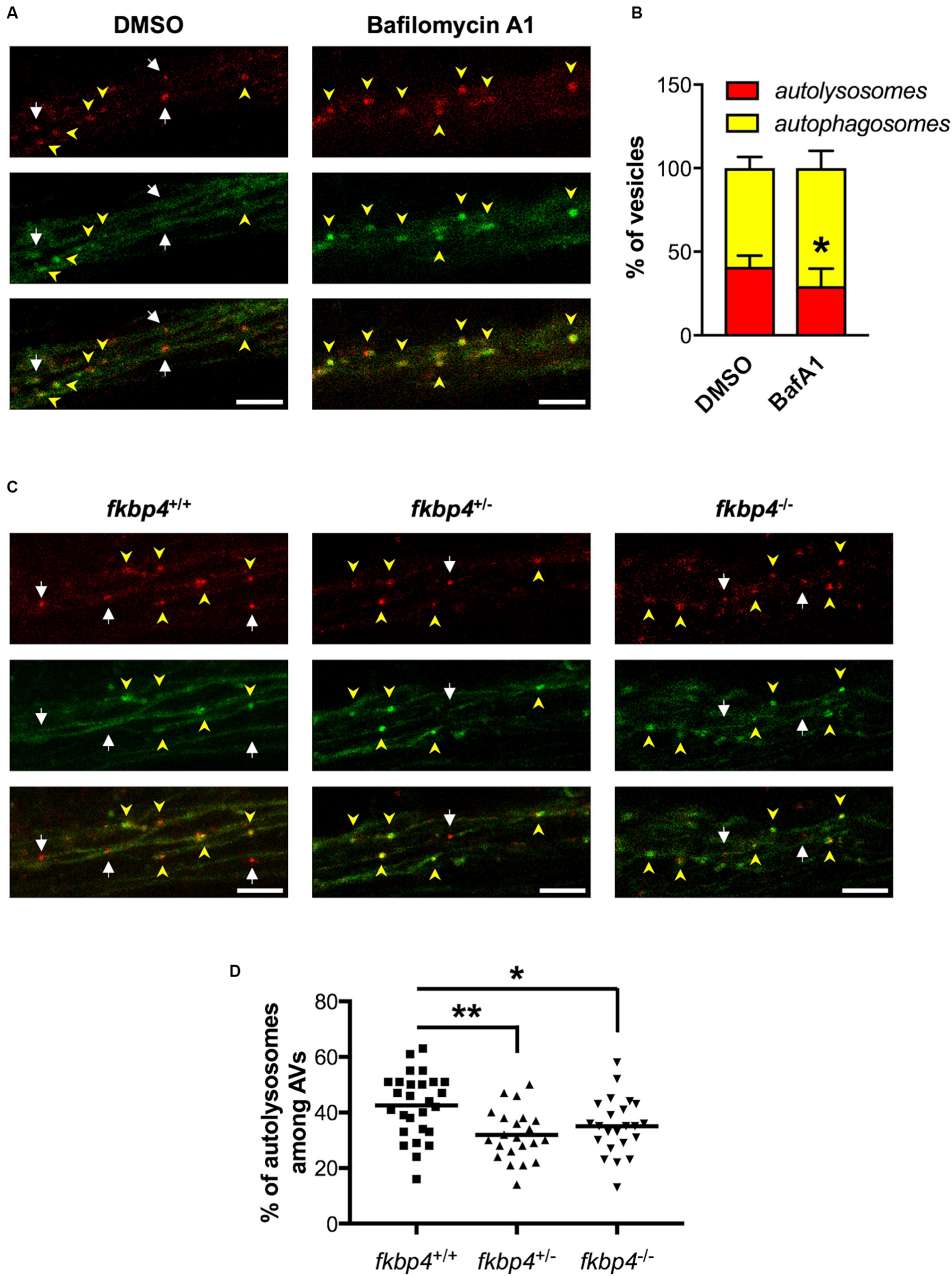
The maturation of autophagosomes is impaired in *fkbp4* mutants. **(A,C)** Representative *z*-projections of the hindbrain region were Lc3-positive vesicles were counted in *HCGL* transgenic fish, either treated with DMSO or Bafilomycin A1 **(A)** or crossed with *fkbp4* mutants **(C)**. Top panels: mCherry; middle panels: EGFP; bottom panels: merged of top and middle panels. Autophagosomes have both red and green signal (yellow arrowheads) whereas autolysosomes show red only signal (white arrows). Scale bar, 5 μm **(B)** Quantification of the percentage of autolysosomes and autophagosomes relative to the total number of autophagic vacuoles in DMSO treated (*n* = 5 embryos) and Bafilomycin A1 treated *HGCL* embryos (*n* = 8 embryos) *HCGL* embryos from one experiment representative of 3 (*t*-test with Welch’s correction, *p* = 0.0332). **(D)** Quantification of the percentage of autolysosomes relative to the total number of autophagic vacuoles in control *fkbp4*^+/+^/*HCGL*^+^ (*n* = 27), *fkbp4*^+/−^/*HCGL*^+^ (*n* = 22, **p = 0.0021 compared to control) and *fkbp4*^−/−^/*HCGL*^+^ (*n* = 23 embryos, *p = 0.0373 compared to control) embryos from 3 independent experiments (One-way ANOVA with Tukey’s multiple comparison). Results are shown as mean ± SD.

To validate the use of this new transgenic line to study autophagic flux, we treated *HCGL* embryos with Bafilomycin A1, a known lysosomal inhibitor that blocks the fusion of autophagosomes with lysosomes (Fodor et al., 2017). A specific region within the hindbrain showing axonal projections rich in Lc3-positive vesicles was selected to count the number of yellow immature versus red mature AVs (Supplementary Figures S2B–D). As expected, the percentage of mature autolysosomes relative to the total Lc3-positive vesicles was significantly decreased in Bafilomycin-treated *HCGL* embryos compared to control DMSO-treated ones (Figures 3A,B).

Transgenic *HCGL* fish were then crossed with the *fkbp4* mutants to compare the autophagic flux between *fkbp4* mutant and WT embryos. For the studied region, we found an average of 42% ± 12% of autolysosomes relative to total AVs in *fkbp4*^+/+^ embryos. This figure was significantly decreased to 32% ± 9% in *fkbp4*^+/−^ and 35% ± 10% in *fkbp4*^−/−^ mutant embryos (Figures 3C,D). Thus, mature acidic autolysosomes appeared to be less abundant in *fkbp4* mutant embryos, with a slightly sharper decrease in heterozygous rather than homozygous *fkbp4* mutants in comparison to WT.

### The kinetic of pathogenic A152T-tau clearance is reduced in *fkbp4*^+/−^ mutants

It has been shown that the clearance of A152T-Tau relies solely on the ALP since the proteasome activity was suppressed in A152T-Tau larvae. Moreover, the clearance of A152T-Tau was slowed following inhibition of lysosomes with ammonium chloride and conversely accelerated by pharmacological induction of autophagy using clonidine or rilmenidine (Lopez et al., 2017).

**Figure 4:**
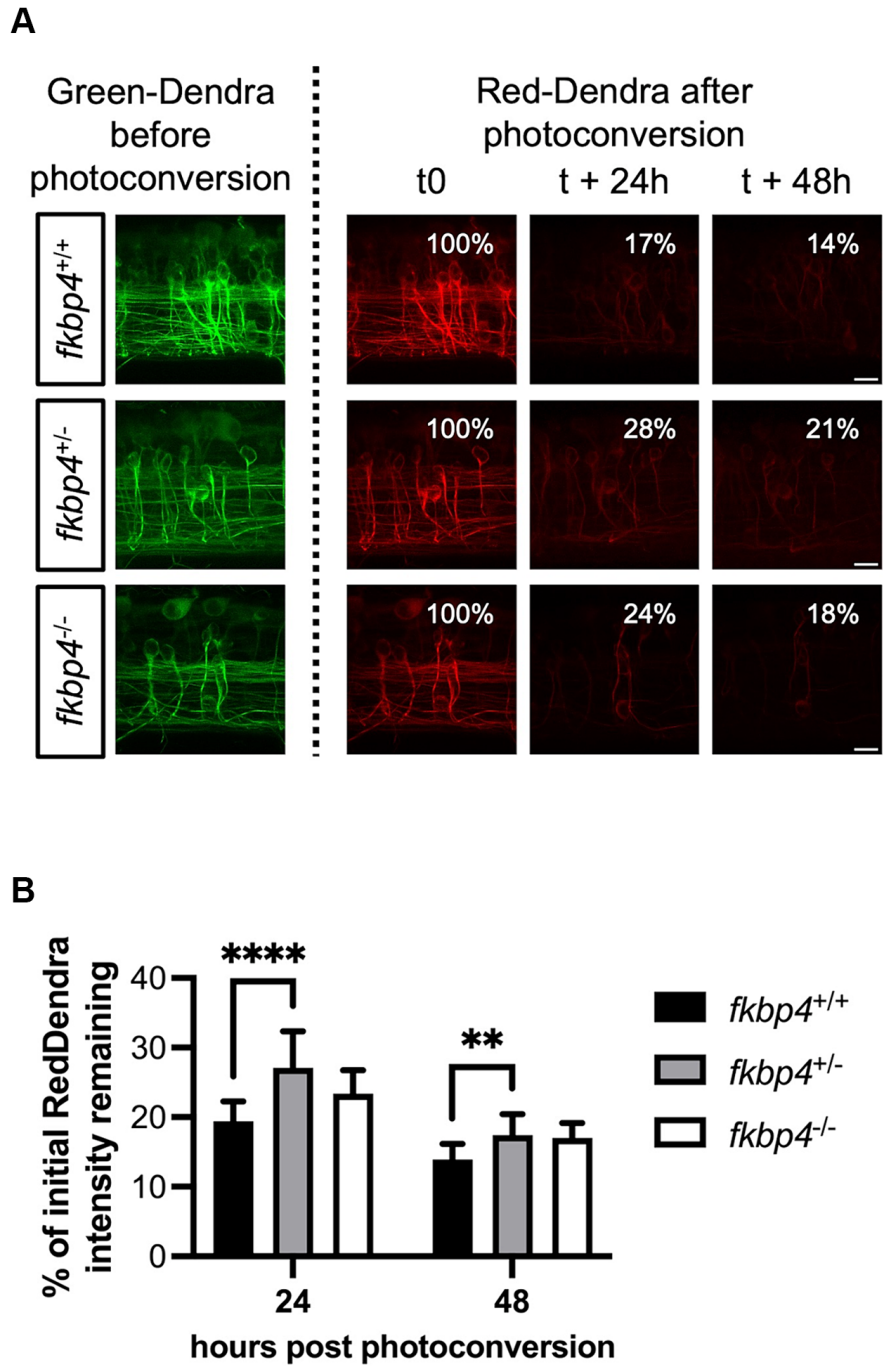
The clearance of A152T-Tau is significantly slower in *fkbp4*^+/−^ mutants. **(A)** Representative images showing *z*-stack projections of the photoconverted neurons with Green-Dendra signal just before photoconversion (left panels) and Red-Dendra signal just after photoconversion, 24 h and 48 h later (right panels) in *fkbp4*^+/+^, *fkbp4*^+/−^, and *fkbp4*^−/−^ embryos expressing Dendra-Tau A152T. Scale bar, 10 μm. **(B)** The remaining intensity of the initial Red-Dendra signal was calculated as percentage of the initial intensity 24 h and 48 h post photoconversion in *fkbp4*^+/+^, *fkbp4*^+/−^, and *fkbp4*^−/−^ larvae with similar A152T-Tau expression (*fkbp4*^+/+^: *n* = 16, average expression 143 ± 31; *fkbp4*^+/−^: *n* = 11, average expression 141 ± 26; *fkbp4*^−/−^: *n* = 5, average expression 141 ± 47) from 4 independent experiments (*fkbp4*^+/−^ vs. *fkbp4*^+/+^ at 24 h: ****p < 0.0001; *fkbp4*^+/−^ vs. *fkbp4*^+/+^ at 48 h: **p = 0.0012; two-way ANOVA with Tukey’s multiple comparison). Results are shown as mean ± SD.

We reasoned that a reduced proportion of mature autolysosomes in *fkbp4* mutant larvae could hinder the clearance of A152T-Tau. To test this hypothesis, we took advantage of the photoconvertible protein Dendra fused to A152T-Tau in the *Tg(UAS::Dendra-Tau A152T)* line to monitor the kinetics of Tau clearance *in vivo*. A specific region of the spinal cord in *fkbp4* mutant and WT embryos with matched expression of A152T-Tau was photoconverted at 48 hpf and the decrease in the red fluorescence was assessed 24 h and 48 h later. *fkbp4*^+/+^ larvae showed a mean of 19.4% ± 2.9% of the initial Red-Dendra intensity remaining 24 h after photoconversion that further decreased to 13.9% ± 2.2% 48 h after photoconversion. This kinetic was not significantly different in *fkbp4*^−/−^ larvae, with a mean of 23.4% ± 3.4 and 17.0% ± 2.2% of the initial Red-Dendra intensity, respectively, remaining 24 h and 48 h after photoconversion. However, these values were significantly increased in *fkbp4*^+/−^ larvae compared to *fkbp4*^+/+^ controls at both time points (27.1% ± 5.2% at 24 h and 17.4 ± 3.0% at 48 h), indicating that the clearance of A152T-Tau was significantly slower in heterozygous fish (Figure 4).

## Discussion

The proper regulation of autophagy is essential for maintaining cellular homeostasis and many studies have implicated autophagy dysregulation in the development of neurodegenerative diseases (Fujikake et al., 2018). By understanding the distinct functions of autophagy in the nervous system and its main regulators, we could better understand how to manipulate autophagy for therapeutic benefit (Djajadikerta et al., 2020). We have previously shown that FKBP52 co-localizes with autophagy-lysosomal markers and an early pathological Tau isoform in AD neurons (Meduri et al., 2016), and that FKBP52 knock down impaired the lysosomal clustering induced by Tau proteotoxicity *in vitro* (Chambraud et al., 2021). Based on these results, we hypothesized that Fkbp52 could impact on physiological lysosomal activity and the autophagy dependent, pathological A152T-Tau clearance.

To test our hypothesis, we took advantage of the translucent zebrafish larvae to follow the dynamic of ALP vesicles in a live organism. It is known that FKBP52, through its interaction with the dynein complex, is required for the nuclear translocation of glucocorticoid receptor (GR) (Wochnik et al., 2005), and can drive the retrograde transport of different cargos along microtubules (Silverstein et al., 1999; Galigniana et al., 2004; Jeong et al., 2016). Consequently, FKBP52 could contribute to the retrograde transport of lysosomes also known to be orchestrated by dynein. To test this, we monitored the movement of Lamp1 vesicles along axons of zebrafish spinal cord, expecting a less processive retrograde transport in the absence of Fkbp52. Surprisingly, we found that the retrograde velocity of Lamp1 vesicles was significantly increased in *fkbp4* mutant zebrafish. Lamp1-positive vesicles in retrograde movement are in fact a heterogenous pool of organelles with different velocities, namely lysosomes, late endosomes, and autolysosomes, that could be differently affected by Fkbp52 deficiency. Indeed, maturating autolysosomes could be part of the subpopulation of Lamp1 vesicles moving retrogradely with a relatively low velocity, as suggested by Lie et al. (2021). Accordingly, the reduced proportion of autolysosomes along axons of *fkbp4* mutants could lead to an overrepresentation of faster moving organelles in comparison to controls, resulting in a biased increase of the mean velocity.

Our work also provides insights into the role of Fkbp52 in autophagic flux *in vivo*. We here generated the Tg(*HuC:mCherry-EGFP-map1lc3B*) line to monitor the autophagic flux within neurons. By analyzing the autophagosomes/autolysosomes ratios along axons of *HCGL* fish, we observed a defective maturation of AVs in *fkbp4* mutants, consistent with our previous results in cultured neuronal cells (Chambraud et al., 2021). Although the underlying mechanism remains to be understood, we hypothesize that Fkbp52 may play a role in controlling the fusions of AVs with lysosomes, which are necessary for their maturation, possibly through fine tuning lysosomal trafficking. Alternatively, Fkbp52 could play a more direct role in the maturation process, by facilitating the acidification of AVs. For instance, it has been described that FKBP52 is able to interact with different transient receptor potential (TRP) channels and modulate their activities in cultured cells (Gkika et al., 2006; Shim et al., 2009). Along the same vein, FKBP52 might modulate the activity of the mucolipin TRP channel which plays a critical role in lysosome acidification and membrane fusion events with late endosomes and AVs (Soyombo et al., 2006; Dong et al., 2008; Li et al., 2013).

Together, our results indicate that Fkbp52 deficiency results in a significant decrease in the percentage of mature autolysosomes being transported to the neuronal cell body. This likely impairs the autophagic degradation of toxic proteins, since we observe a reduced clearance of A152T-Tau, known to be degraded by autophagy (Lopez et al., 2017; Caballero et al., 2018), in *fkbp4* heterozygous mutant. This result is consistent with our previous finding whereby a decrease in FKBP52 induces an accumulation of P301S-Tau in dorsal root ganglia neurons from mice under long-term proteotoxic stress (Chambraud et al., 2021).

It is important to note that the defects observed in heterozygous *fkbp4* mutants are globally enhanced in comparison to homozygous ones. This raises the possibility of a genetic compensation in embryos lacking Fkbp52. The non-sense mediated decay of mutant *fkbp4* mRNA could activate a compensatory network, possibly members of the Fkbp family and/or other proteins, leading to a milder phenotype, similar to other knockout zebrafish mutants (Rossi et al., 2015; Zhu et al., 2017; El-Brolosy et al., 2019; Diofano et al., 2020). Indeed, the mRNA levels of *fkbp5* (coding for Fkbp51), known to be functionally antagonistic to Fkbp52 in some instances (Wochnik et al., 2005), are significantly reduced in *fkbp4*^−/−^ mutants in comparison to heterozygous mutants (Supplementary Figure S3). Whether these two proteins act as antagonists in this particular context is still to be determined.

Interestingly, we observed no obvious defects in *fkbp4*^+/−^ adult fish whereas *fkbp4*^−/−^ adult males were mostly sterile. This phenotype is unlikely to be linked to the function of Fkbp52 in the ALP, but rather to its role as a cochaperone of steroid hormone receptors. Indeed, male FKBP52 knock out mice show several defects in their reproductive tissues that are linked to androgen receptor insensitivity (Cheung-Flynn et al., 2005; Chen et al., 2010). We repeatedly observed that most of the *fkbp4*^−/−^ males do not display the expected courtship behavior during mating, consistent with the phenotype described upon genetic disruption of androgen signaling in zebrafish (Yong et al., 2017; Shu et al., 2020).

Altogether, our findings reveal an important role for Fkbp52 in the transport of lysosomes, the maturation of autophagic vacuoles and the clearance of pathogenic Tau proteins *in vivo*. Therefore, the reduced levels of FKBP52 in the brains of AD or FTLD-Tau patients might contribute to the accumulation of toxic proteins by impairing autophagy, further worsening the pathogenesis of the disease. Preventing FKBP52 decrease or restoring its lost function in affected neurons might represent a new potential therapeutic approach to slow down progression of Tau pathology.

## Data availability statement

The raw data supporting the conclusions of this article will be made available by the authors, without undue reservation.

## Ethics statement

The animal studies were approved by DDPP Val de Marne, France, under license number F 94-043-013. The studies were conducted in accordance with the local legislation and institutional requirements. Written informed consent was obtained from the owners for the participation of their animals in this study.

## Author contributions

EL: Conceptualization, Formal analysis, Investigation, Methodology, Supervision, Writing – original draft, Writing – review & editing, Visualization. LC: Investigation, Methodology, Writing – review & editing. MG: Investigation, Methodology, Writing – review & editing. E-EB: Funding acquisition, Project administration, Writing – review & editing. MT: Conceptualization, Funding acquisition, Investigation, Supervision, Writing – review & editing. JG: Conceptualization, Formal analysis, Funding acquisition, Investigation, Supervision, Writing – review & editing.

## Footnotes

^1^ https://zfin.org/zf_info/zfbook/cont.html

^2^ crispor.tefor.net
