# Supplementary material for: A decrease in Fkbp52 alters autophagosome maturation and A152T-tau clearance in vivo
Source: Front Cell Neurosci. 2024 Jul 25;18:1425222. doi: 10.3389/fncel.2024.1425222 (PMC11306173; doi:10.3389/fncel.2024.1425222)
Supplement: Supplementary file 1 [file Table_1.DOCX]

**TABLE S1**. Primers used in qPCR

| **Gene** | **Forward primer sequence (5'-3’)** | **Reverse primer sequence (5'-3’)** |
| --- | --- | --- |
| *fkbp4* | CACTCTTCACCATCAAACCAAAGT | CCCAGGATTCTTTGGCCTTT |
| *fkbp5* | GGGAAATGGACCTCAAAGAGA | CGATCCGCTGGTACTGAATTA |
| *fkbp1aa* | TCATATTCGACGTGGAGCTGATT | CAGAGCTAAGCTAAGTGGTATCGCTATT |
| *fkbp1ab* | GGGAGTGGAAATCGAGACCATA | CATCTGTCAGGGAGCCAACATAA |
| *elFA* | CTTCTCAGGCTGACTGTGC | CCGCTAGCATTACCCTCC |
