# Supplementary material for: A decrease in Fkbp52 alters autophagosome maturation and A152T-tau clearance in vivo
Source: Front Cell Neurosci. 2024 Jul 25;18:1425222. doi: 10.3389/fncel.2024.1425222 (PMC11306173; doi:10.3389/fncel.2024.1425222)
Supplement: Supplementary file 2 [file Table_2.docx]

Supplementary Material

## SUPPLEMENTARY FIGURE 1. Retrograde axonal transport is also altered in *fkbp4* Δ11 mutant.

**(A)** Alignment of the CRISPR/Cas9 targeted sequence of the Danio rerio *fkbp4* gene in WT and Δ11 mutant. Light green: gRNA target sequence. Dark green: PAM sequence. The 11bp deletion induces a frameshift and a premature stop codon (orange). **(B)** Structural domains of the WT and Δ11 Fkbp52 proteins. FK1: peptidyl-prolyl isomerase (PPIase) domain. FK2: PPIase-like domain. TPR: tetratricopeptide repeat domain. CaM: putative calmodulin binding site. **(C)** Representative immunoblot and resulting quantification showing Fkbp52 protein levels in WT*, fkbp4* ^Δ11/WT^ and *fkbp4* ^Δ11/Δ11^ embryos. Tubulin was used as loading control. **(D)** Quantification of anterograde and retrograde velocity of Lamp1-positive vesicles along axons of spinal cord in WT (n = 19), *fkbp4* ^Δ11/WT^ (n = 13) and *fkbp4* ^Δ11/Δ11^ (n = 14) embryos from 4 independent experiments. Analysis includes ~ 12 tracked Lamp1 vesicles per embryo (Kruskal-Wallis, Dunn’s multiple comparisons test). Results are shown as mean ± SEM.

## SUPPLEMENTARY FIGURE 2. The *Tg(HuC::mCherry-EGFP-map1lc3b)* line allows the visualization of autophagosomes and autolysosomes in live neurons.

**(A)** Z-projections of confocal live imaging in 48 hpf HCGL embryos showing red vesicles and a diffuse green cytoplasmic signal in neuronal cell bodies of the spinal cord and brain. **(B)** Schematic of a 48 hpf embryo. The red boxed area shows the hindbrain region where Lc3-positive vesicles were counted along axons. **(C)** Z-projections of confocal live imaging in 48 hpf HCGL embryos showing vesicles along axons in the hindbrain region depicted in B. **(D)** Magnification of the boxed area in C presented as a z-projection (top image) and as single optical slices (bottom images) with the corresponding profile plots of mCherry and EGFP signals along the segmented line drawn on the top image. Yellow arrowheads show autophagosomes (red and green signal) and white arrows show autolysosomes (red only signal). Scale bar, 10 µm.

**SUPPLEMENTARY FIGURE 3. Down-regulation of *fkbp5* expression in *fkbp4*^-/-^ mutants compared to *fkbp4*^+/-^ siblings.**

**(A-D)** RT-qPCR results. The mRNA levels of *fkbp4* are sharply decreased in both *fkbp4*^-/-^ and *fkbp4^+^*^/-^ embryos compared to WT controls **(A)** whereas *fkbp5* expression is significantly reduced in *fkbp4*^-/-^ mutants compared to *fkbp4^+^*^/-^ siblings **(B)**. Expression of both *fkbp1aa* and *fkbp1ab* are unaltered in *fkbp4* mutants **(C-D)**.

## SUPPLEMENTARY VIDEO 1. Real-time imaging of Lamp1-GFP vesicles in a wild-type embryo at 48 hpf.

A 48 hpf embryo expressing Lamp1-GFP along axons of spinal cord after *pHuC-Lamp1-GFP* injection; the embryo was imaged every 650 milliseconds for several minutes by confocal microscopy. This video represents ~80 seconds of continuous real-time imaging. White arrows highlight an anterograde moving Lamp1-GFP vesicle while red arrowheads highlight a retrograde moving vesicle.

## SUPPLEMENTARY VIDEO 2. Real-time imaging of Lamp1-GFP vesicles in a *fkbp4^+/-^* embryo at 48 hpf.

A 48 hpf embryo expressing Lamp1-GFP along axons of spinal cord after *pHuC-Lamp1-GFP* injection; the embryo was imaged every 650 milliseconds for several minutes by confocal microscopy. This video represents ~40 seconds of continuous real-time imaging. White arrows highlight an anterograde moving Lamp1-GFP vesicle while red arrowheads highlight a retrograde moving vesicle.

## SUPPLEMENTARY VIDEO 3. Real-time imaging of Lamp1-GFP vesicles in a *fkbp4^-/-^* embryo at 48 hpf.

A 48 hpf embryo expressing Lamp1-GFP along axons of spinal cord after *pHuC-Lamp1-GFP* injection; the embryo was imaged every 650 milliseconds for several minutes by confocal microscopy. This video represents ~40 seconds of continuous real-time imaging. White arrows highlight an anterograde moving Lamp1-GFP vesicle while red arrowheads highlight a retrograde moving vesicle.
