# Supplementary figures and images for: A decrease in Fkbp52 alters autophagosome maturation and A152T-tau clearance in vivo
Source: Front Cell Neurosci. 2024 Jul 25;18:1425222. doi: 10.3389/fncel.2024.1425222 (PMC11306173; doi:10.3389/fncel.2024.1425222)

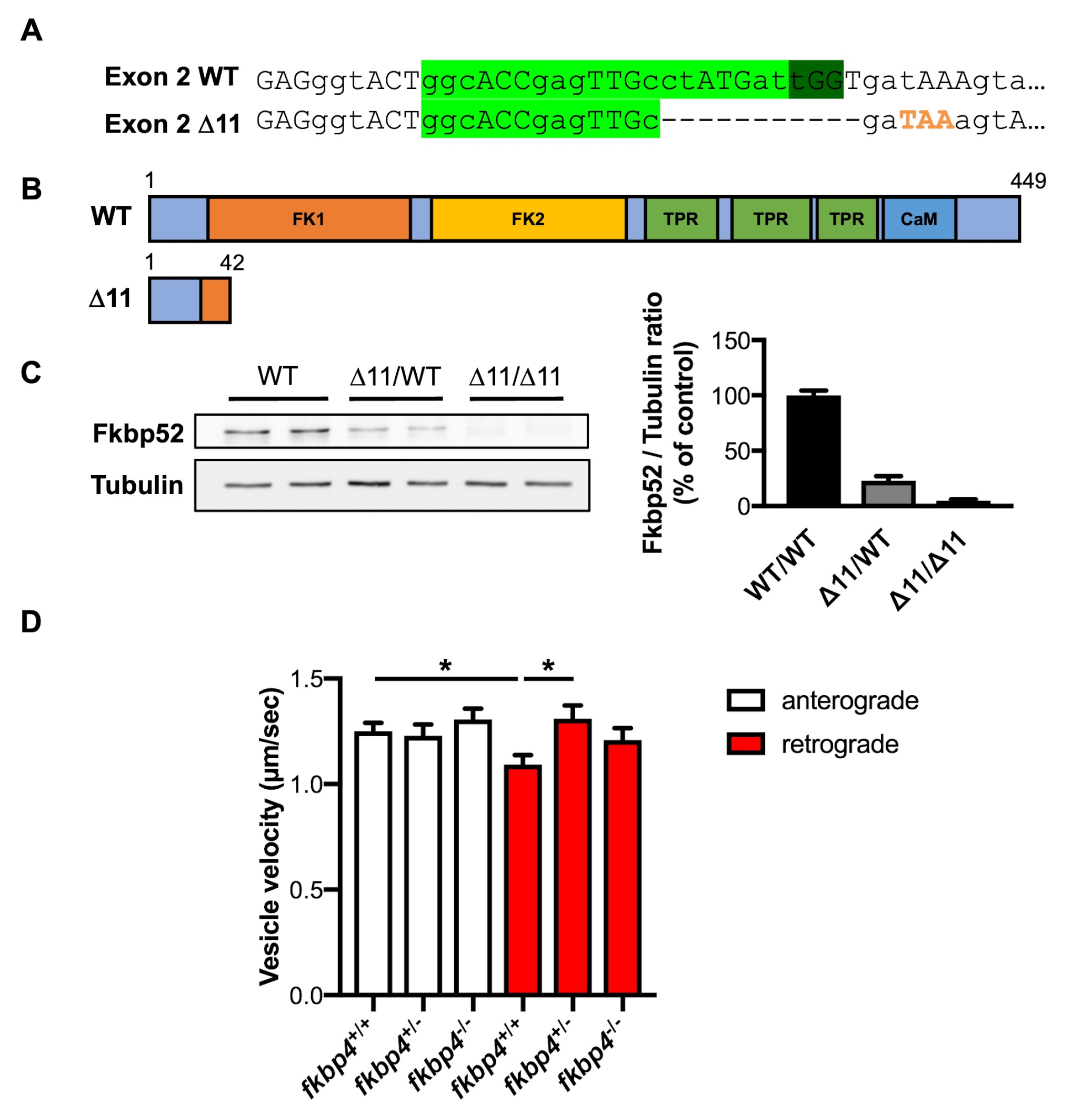

Supplement: Supplementary file 6 [file Image_1.TIF]

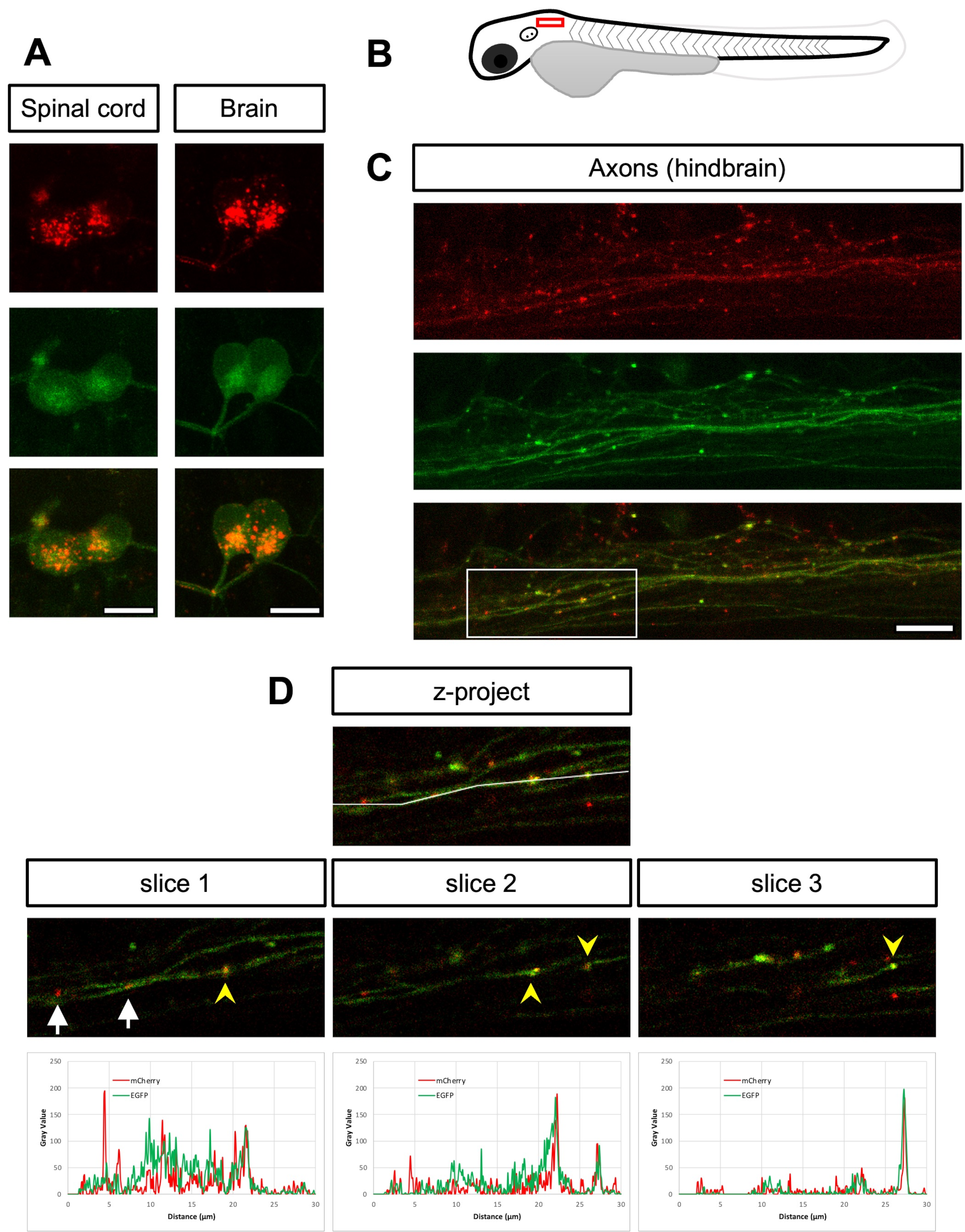

Supplement: Supplementary file 7 [file Image_2.TIF]

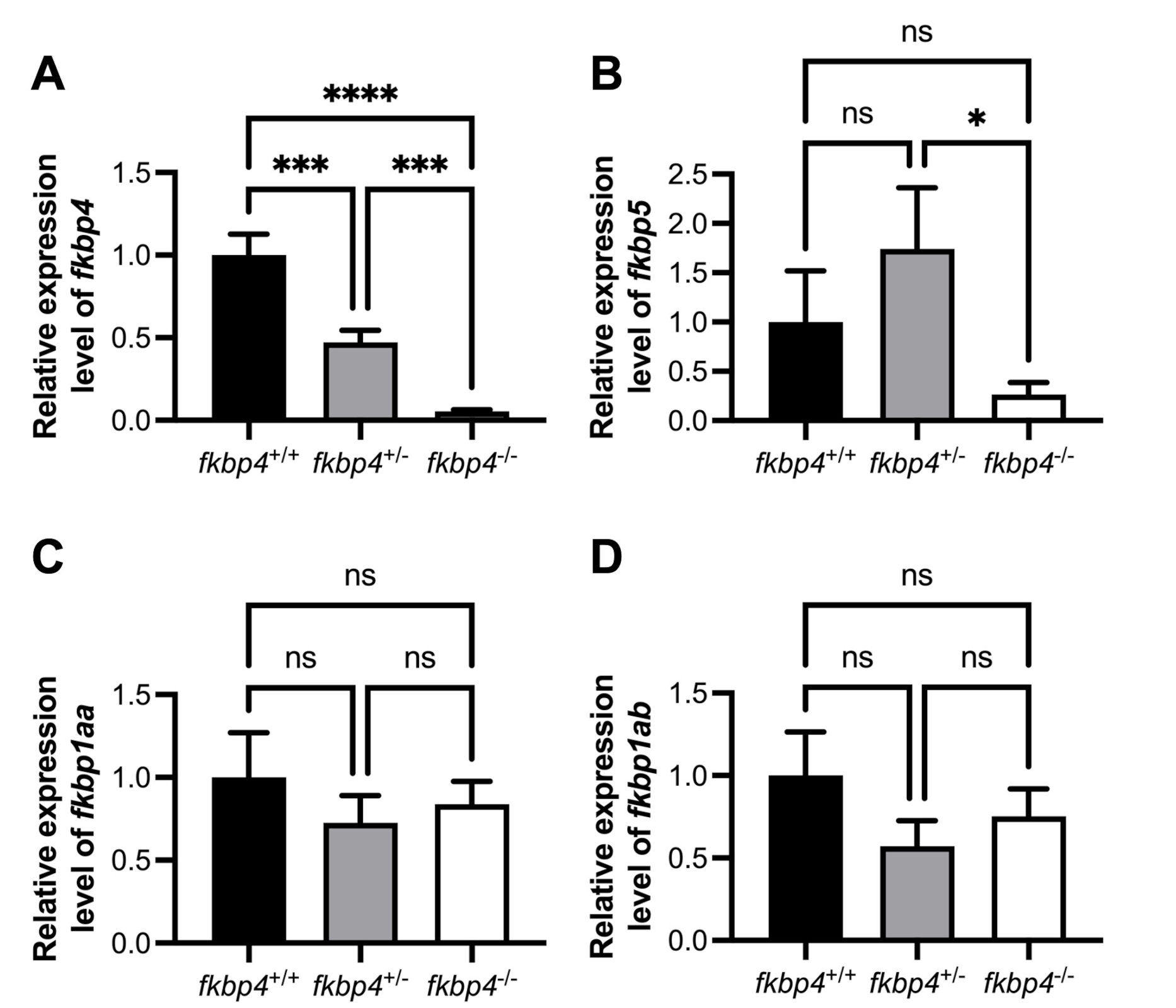

Supplement: Supplementary file 8 [file Image_3.TIF]
